# Diagnostic Utility of New SCAT5 Neurological Screen Sub-tests

**DOI:** 10.1186/s40798-021-00303-z

**Published:** 2021-02-15

**Authors:** Gordon Ward Fuller, John Miles, Ross Tucker, Marc Douglas, Martin Raftery, Eanna Falvey, Prabhat Mathema

**Affiliations:** 1 Centre for Urgent and Emergency Care Research, School of Health and Related Research, University of Sheffield, Regent Court, 30 Regent Street, Sheffield, S1 4DA UK; 2 Wales Rugby Union, Principality Stadium, Westgate Street, Cardiff, CF10 1NS UK; 3 University of Cape Town School of Management Studies, Cape Town, South Africa; 4 World Rugby, World Rugby House 8-10 Lower Pembroke Street, Dublin 2, Ireland

**Keywords:** Brain Concussion, Screening, Rugby, Diagnostic accuracy, SCAT5

## Abstract

**Background:**

The Sports Concussion Assessment Tool (SCAT) is recommended to screen for concussion following head impact events in elite sport. The most recent 5th edition (SCAT5) included a ‘rapid neurological screen’ which introduced new subtests examining comprehension, passive neck movement, and diplopia. This study evaluated the additional diagnostic value of these new subtests.

**Methods:**

A prospective cohort study was performed in the Pro14 elite Rugby Union competition between September 2018 and January 2020. The SCAT5 was administered by the team doctor to players undergoing off-field screening for concussion during a medical room assessment. Sensitivity, specificity, false negatives, and positives were examined for SCAT5 comprehension, passive neck movement, and diplopia subtests. The reference standard was a final diagnosis of concussion, established by serial standardised clinical assessments over 48 h.

**Results:**

Ninety-three players undergoing off-field screening for concussion were included. Sensitivity and specificity of the comprehension, passive neck movement, and diplopia subtests were 0, 8, 5% and 0, 91, 97%, respectively (concussion prevalence 63%). No players had any abnormality in comprehension. No players had abnormal passive neck movement or diplopia in the absence of abnormalities in other SCAT5 sub-components.

**Conclusions:**

The new SCAT5 neurological screen subtests are normal in the majority of players undergoing off-field concussion screening and appear to lack diagnostic utility over and above other SCAT5 subtests.

## Key Points

- The Sports Concussion Assessment Tool 5th edition (SCAT5) includes a ‘rapid neurological screen’ with new subtests examining comprehension, passive neck movement, and diplopia.
- In elite Rugby Union players undergoing off-field concussion screening, no players had any abnormality in comprehension; or had diplopia, or painful passive neck movement, in the absence of abnormalities in other SCAT5 sub-components.
- The neurological screen subtests did not affect the diagnostic accuracy of the SCAT5 and did not detect any additional concussed players, or independently result in any false-positive cases.

## Background

Concussion is a common injury in collision sports [1]. Given the diverse array of presenting symptoms and signs that can occur following concussion, a standardised multi-modal diagnostic approach has been recommended [2]. The International Consensus Conference for Concussion in Sport therefore developed the Sports Concussion Assessment Tool (SCAT) to screen for suspected concussion, standardise evaluation of sports-related concussion, track player recovery, and serve as a tool for player education [2].

Development of the SCAT has been an iterative process. The latest version, the SCAT5, included a ‘rapid neurological screen’, adding new subtests evaluating comprehension, passive neck movement and diplopia. Although the rationale for the new sub-tests was not fully elaborated, the authors state that they will ‘increase the utility of the tool’ [2]. The performance of other SCAT components has been previously evaluated in detail [3]; however, there is an absence of evidence for the diagnostic accuracy of the newer neurological subtests in off-field screening for concussion [4].

This study therefore aimed to determine if the new SCAT5 neurological screen subtests are effective additions to the SCAT in off-field screening for sport-related concussion. Specific objectives were to describe the distribution of subtest results; evaluate diagnostic accuracy in Rugby Union players undergoing off-field concussion screening; and describe the additional value compared to other SCAT subtests.

## Methods

### Study Design and Population

A prospective cohort study was conducted in the male, adult, elite-level Rugby Union ‘PRO14’ competition players over two seasons between September 2018 and January 2020. The source population consisted of players from Scottish (2 teams), Irish (4), South African (2), Welsh (4), and Italian (2) elite level rugby union teams. The study population comprised players undergoing off-field concussion screening with the SCAT5 after identification of a meaningful head impact event with the potential to cause concussion during competitive PRO14 matches.

### Procedures

The World Rugby Head Injury Assessment (HIA) process for management of head impacts during competitive matches has been described previousl y[3]. Briefly, players demonstrating clear signs of concussion (e.g. loss of consciousness, tonic posturing, ataxia, seizures) are immediately and permanently removed from the remainder of the match, without undergoing further off-field concussion screening. Players sustaining head impacts where the consequences were unclear, e.g. dangerous mechanism, undergo an off-field concussion screening assessment (termed the HIA-1 assessment). The assessment is conducted by the team doctor in a medical room during a 15-min player interchange. In this study, off-field screening was performed using the SCAT5 instrument, with results interpreted in comparison to pre-season player-specific baseline results. All players entering the HIA process receive repeat clinical assessments made by the team doctor post-match (termed the HIA-2 assessment) and after 48 h rest (termed the HIA-3 assessment), supported by cognitive assessments (typically a computerised neuro-cognitive tool such as CogSport) [5].

### Index Tests and Reference Standard

The index tests under consideration were the component subtests of the new ‘rapid neurological screen’ in the SCAT5, performed during off-field concussion HIA-1 screening assessments, namely: comprehension (normal = ability to read aloud and follow instructions without difficulty), passive neck movement (normal = full range of pain-free passive cervical spine movement), and diplopia (normal = no diplopia in any plane of eye movement). Subtest values were interpreted in comparison to player-specific pre-season baseline SCAT5 results.

The reference standard, against which performance of each index test was compared, was a clinical diagnosis of concussion following completion of the HIA process.

### Data Collection

HIA process data are routinely recorded at the point of assessment by physicians using a tablet based, web-hosted platform, with standardised data collection forms (CSx Systems, Auckland, New Zealand) [6]. Any missing CSx outcome data was collected by direct communication with team doctors.

### Analysis

The analysis proceeded in three stages. Firstly, player flow through the study and demographic characteristics were described. Secondly, the proportion of abnormal index test results was described overall, and separately for concussed/non-concussed players. Thirdly, the sensitivity and specificity were calculated for each index test against the reference standard of a final clinical diagnosis of concussion. Finally, the additional value of each subtest, over and above other SCAT5 components, was investigated by examining extra false positives (non-concussed players with abnormal index test findings, but otherwise normal SCAT5 subtest results) and extra true positives (concussed players with abnormal index test finding, but otherwise normal SCAT5 subtest results).

### Sample Size, Statistics, Ethics, and Funding

A census sample of head impact events undergoing off-field HIA-1 concussion screens over two PRO14 seasons were included. The width of confidence intervals indicates the precision of results. Statistical analyses were carried out in Stata version 13.1 (StataCorp., College Station, USA). Available case analyses were performed with a conventional significance level (α) of 0.05 used. The unit of analysis was consecutive significant head impact events, and individual players could be included in the sample more than once if subject to recurrent HIA-1 concussion screens. Ethical approval was provided for analysis of the data from the University of Sheffield. All players provided informed consent for use of data prior to the start of the season. All data were anonymised. The study was not funded.

## Results

**Fig. 1:**
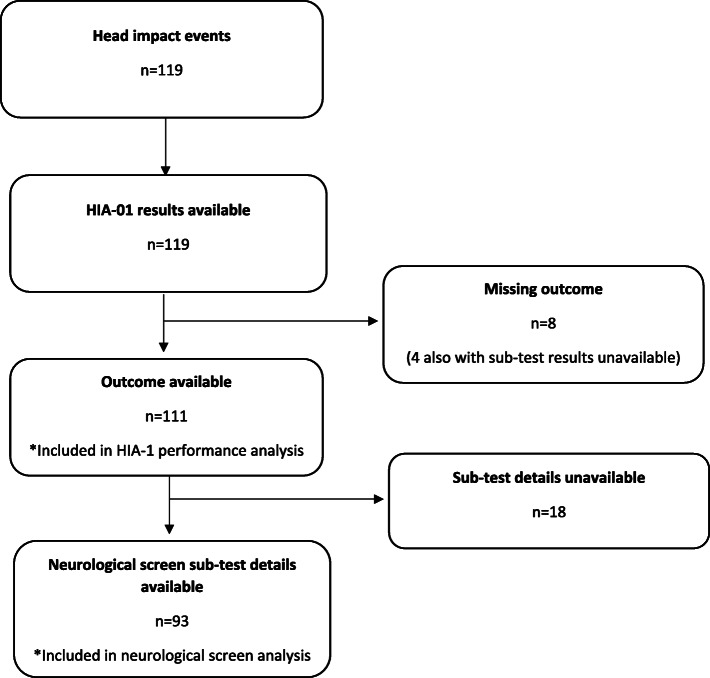
Derivation of the study sample

A total of 119 head impact events, where the consequences were unclear, were detected in 107 players who underwent off-field concussion screening. Overall SCAT5 HIA-1 screening assessment results were recorded for all players, with outcome data absent in 8 players. SCAT5 sub-test results were unavailable for a further 22 players. A flow chart describing derivation of the study sample is presented in Fig. 1. The median age was 28 years (Interquartile range 25–31) with 79% of players having sustained a previous career concussion (median 2, interquartile range 1–3).

Overall, 64 players undergoing off-field screening were diagnosed with concussion, giving a prevalence of 57.6% (*n* = 111). Of these concussed players, 57 were correctly removed from play following their HIA-1 assessment. Of the non-concussed players, 38/47 were correctly returned to play. Sensitivity and specificity of the SCAT5 HIA-1 off-field screening assessment were therefore 89.1% (95%CI 78.8–95.5%) and 80.9% (66.7–90.9%, *n* = 111) respectively.

New neurological screen subtests were normal in the majority of players undergoing off-field concussion screening. No players had any abnormality in comprehension; passive neck movement was painful in 8 players (8.6%); and 4 cases had diplopia (4.3%, *n* = 93). There were no significant differences in the proportion of concussed v non-concussed players with painful passive neck movement or diplopia (*p* = 0.9 and 0.6, *n* = 93). Sensitivity and specificity of the comprehension, passive neck movement, and diplopia subtests were 0, 8, 5% and 0, 91, 97%, respectively (concussion prevalence 63.4% *n* = 93). Table 1 summarises the results and diagnostic accuracy of new SCAT5 neurological screen subtests.

**Table 1:** Results and diagnostic accuracy of new SCAT5 neurological screen subtests

***N*** = 93	Proportion with abnormal result (***n***, %)			***p*** value	Diagnostic accuracy		Contribution to SCAT5 performance^**a**^	
Neurological screen subtest	Overall (***n*** = 93)	Concussed (***n*** = 59)	Non-concussed (***n*** = 34)		Sensitivity (%)	Specificity (%)	Additional true positives	Additional false positives
					(95% CI)			
Comprehension	0 (0)	0 (0)	0 (0)	–	–	–	0	0
Passive neck movement	8 (8.6)	5 (8.5)	3 (8.8)	0.9	8.5 (2.8–18.7)	91.2 (76.2–98.1)	0	0
Diplopia	4 (4.3)	3 (5.1)	1 (2.9)	0.6	5.1 (1.1–14.1)	97.1 (84.7–99.9)	0	0

^a^If SCAT5 subtests interpreted according to baseline results

No players’ painful passive neck movement or diplopia in the absence of abnormalities in other SCAT5 sub-components compared to baseline values. The passive neck movement and diplopia subtests therefore did not affect the diagnostic accuracy of the SCAT5, and did not detect any additional concussed players, or independently result in any false-positive cases.

## Discussion

New subtests included in the SCAT5 rapid neurological screen were normal in the majority of players undergoing off-field concussion screening. Sensitivity and specificity of the comprehension, passive neck movement, and diplopia subtests were 0, 8, 5% and 0, 91, 97%, respectively. The new neurological screen subtests did not affect the diagnostic accuracy of the SCAT5. No players had any abnormality in comprehension, or had diplopia, or painful passive neck movement, in the absence of abnormalities in other SCAT5 sub-components.

The SCAT5 is the fourth iteration of the Sports Concussion Assessment Tool [2]. The original version had a ‘neurological screening’ domain, including subtests similarly examining speech and eye motion [7]. However, these were subsequently removed from the SCAT2 and SCAT3 [8, 9], prior to re-introduction in the SCAT5 [2]. ‘Neck pain’ has been part of the symptom checklist in all SCAT editions, with a neck examination introduced from the SCAT3 onwards [9]. However, specific evaluation of full range of pain-free passive cervical spine movement is specific to the SCAT5.

Concussion is considered a subset of mild traumatic brain injury largely reflecting a functional disturbance of brain disturbance [1]. The new SCAT5 neurological subtests of comprehension and diplopia are blunt examinations, which might be expected to be normal in the absence of other grossly abnormal neurological symptoms or signs. The findings that these subtests were largely normal in a population without overt signs of concussion, and only abnormal in conjunction with other SCAT5 subtest abnormalities, may therefore be unsurprising. Previous studies have demonstrated visual problems associated with traumatic brain injury and more detailed neuro-ophthalmological testing might have utility in off-field concussion screening [10].

Pain on passive neck movement is not a traditional neurological examination, and neck pain would not be expected in an isolated functional brain injury. However, neck examination could provide useful information during concussion screening. Concussion is commonly associated with concomitant neck injury including muscle strain, ligamentous sprains or, rarely, arterial dissection or bony injuries, which could confound the detection of concussion [11]. Moreover, despite limited evidence, if the presence of a neck sprain is also predictive of concussion, passive neck movement could potentially be a useful screening test. However in this study, passive neck examination did not appear to add any value over and above other SCAT5 subtests.

The SCAT5 consist of multiple subtests applied concurrently. In this testing paradigm, as further subtests are added, sensitivity will increase and specificity fall. Ideally, to maximise diagnostic accuracy, the optimal combination of individual subtests would be chosen that individually demonstrate reasonable sensitivity to detect new cases of concussion over and above other subtests; but that also have satisfactory specificity to minimise false-positive cases. The performance of individual SCAT components for off-field concussion screening, and their optimal combination, has been examined previously [3]. In the current study, the new comprehension, passive neck movement, and diplopia subtests demonstrated worse performance than other individual SCAT subtests, with no additional value. Their very low sensitivities (0, 8, and 5%, respectively) also indicate that they could not be substituted for other SCAT5 components. These results should be generalizable throughout Rugby and, as the subtests tests are relatively simple, are likely to have external validity in other elite sports. It is essential to note that ‘the diagnosis of concussion relies on a clinical synthesis of complex, non-specific and at times contradictory information’ [2]. It has been shown previously that team doctors often use expert judgement when interpreting off-field screening tests results compared to baseline or normative thresholds [3]. Despite not providing direct information, it is therefore possible that the new neurological subtests provide global information to inform an overall clinical assessment. Importantly, the SCAT5 is also used in other contexts, such as diagnosis or tracking recovery, and the new neurological subtests may be more useful in these applications, which were outside the scope of the current study.

This study has several limitations. Firstly, the sample size is relatively small, although the 95% confidence intervals for sensitivity and specificity are not consistent with a clinically significant ability of new subtests to discriminate concussed players. Secondly, there was missing data in some players which could have introduced selection bias. These were predominantly non-concussed cases with normal HIA-1 off-field screening results (*n* = 17/26), where the neurological sub-tests are highly likely to be normal, suggesting that our results are conservative and are unlikely to have underestimated sensitivity. Thirdly, team doctors administered the SCAT5 in both index test and reference standard assessments, there is a risk of incorporation and diagnostic review bias. Finally, there are no convincing and objective gold standard criteria for the diagnosis of concussion which could lead to outcome misclassification and information bias.

## Conclusions

The new SCAT5 neurological screen subtests are normal in the majority of players undergoing off-field concussion screening and appear to lack diagnostic utility over and above other SCAT5 subtests. If corroborated, our findings suggest that these components might be safely omitted from side-line concussion screening assessments.

## Data Availability

The datasets generated during and/or analysed during the current study are not publicly available due to the terms of athlete consent and ethical approvals. However, anonymised data may be available from the corresponding author on reasonable request.
